# Innate Cells: The Alternative Source of IL-17 in Axial and Peripheral Spondyloarthritis?

**DOI:** 10.3389/fimmu.2020.553742

**Published:** 2021-01-08

**Authors:** Nicolas Rosine, Corinne Miceli-Richard

**Affiliations:** ^1^ Unité Mixte AP-HP/Institut Pasteur, Institut Pasteur, Immunoregulation Unit, Paris, France; ^2^ Paris University, Department of Rheumatology—Hôpital Cochin. Assistance Publique—Hôpitaux de Paris, EULAR Center of Excellence, Paris, France

**Keywords:** spondyloarthritis, psoriatic arthritis, IL-17A, innate cells, IL-23, IL-17

## Abstract

Spondyloarthritis (SpA) is a chronic inflammatory rheumatism characterized by inflammation of sacroiliac joints, peripheral joints, and spine. The Assessment of SpondyloArthritis Society describes three disease forms: axial (axSpA), peripheral, and enthesitic SpA. Each may be associated with extra-articular manifestations: psoriasis, inflammatory bowel disease, and acute anterior uveitis. Genome-wide association studies performed in axSpA and psoriatic arthritis (PsA) have shown a shared genetic background, especially the interleukin 23 (IL-23)/IL-17 pathway, which suggests pathophysiological similarities. The convincing positive results of clinical trials assessing the effect of secukinumab and ixekizumab (anti-IL-17A monoclonal antibodies) in axSpA and PsA have reinforced the speculated crucial role of IL-17 in SpA. Nevertheless, and obviously unexpectedly, the differential efficacy of anti-IL-23–targeted treatments between axSpA (failure) and PsA (success) has profoundly disrupted our presumed knowledge of disease pathogeny. The cells able to secrete IL-17, their dependence on IL-23, and their respective role according to the clinical form of the disease is at the heart of the current debate to potentially explain these observed differences in efficacy of IL-23/IL-17–targeted therapy. In fact, IL-17 secretion is usually mainly related to T helper 17 lymphocytes. Nevertheless, several innate immune cells express IL-23 receptor and can produce IL-17. To what extent these alternative cell populations can produce IL-17 independent of IL-23 and their respective involvement in axSpA and PsA are the crucial scientific questions in SpA. From this viewpoint, this is a nice example of a reverse path from bedside to bench, in which the results of therapeutic trials allow for reflecting more in depth on the pathophysiology of a disease. Here we provide an overview of each innate immunity-producing IL-17 cell subset and their respective role in disease pathogeny at the current level of our knowledge.

## Introduction

Spondyloarthritis (SpA) is a chronic inflammatory disease characterized by inflammation of the sacroiliac joints, peripheral joints, and spine. The Assessment of SpondyloArthritis Society describes three disease forms: axial (axSpA), peripheral, and enthesitic. Each of these forms can be associated with extra-articular manifestations: psoriasis, inflammatory bowel disease, and acute anterior uveitis. Genome-wide association studies performed in the axial and more severe form (i.e., ankylosing spondylitis [AS]) have revealed significant genetic associations with several polymorphisms involved in the T helper 17 cell (Th17) pathway: IL-23 receptor (IL-23R), IL-12B, IL-1R2, IL-6R, and RUNX3. These results, among others, have shed light on the role of the Th17 pathway in axSpA. Of note, GWAS performed in psoriasis and psoriatic arthritis (PsA) have shown similar genetic associations regarding Th17 pathway components, which suggests a common genetic background and potential shared pathogenic mechanisms for this spectrum of diseases.

Th17 cells are the third subset of effector CD4^+^ Th cells characterized by the expression of IL-17. IL-17 contributes to the clearance of a range of pathogens (e.g., *Candida albicans*, *Klebsiella pneumoniae*, and *Staphylococcus aureus*)—the Yin and beneficial face of IL-17. Nevertheless, they have also been associated with the pathogenesis of several immune-mediated inflammatory diseases—the Yang and deleterious face of Th17 cells.

In recent years, the involvement of the IL-23/IL-17 axis in the pathophysiology of SpA has been established both in peripheral blood and affected tissues. Several mouse models have shown the major role of Th17 cells in triggering autoimmunity and autoimmune diseases (1). Synovial fluid and/or serum from patients with active AS, undifferentiated SpA, and PsA show increased expression of IL-17A (2, 3). Consistent with the increased expression of serum IL-17, the increased absolute number and/or frequency of circulating CD4^+^ Th17 cells have been reported in AS, PsA, and reactive arthritis (4–6), but these observations have been controversial (7, 8). Regardless, IL-17 production was found not restricted to CD4^+^ Th17 cells. Other innate immune cells can produce IL-17, and even if some represent a quantitatively minor cell subset, their potential role in SpA pathophysiology should not be underestimated.

## Th17 Cells: The “Classical” IL-17–Secreting Cells

Th17 cells are involved in the defense against certain bacterial and fungal infections, participate in the stimulation and recruitment of polynuclear neutrophil cells, and stimulate the production of antimicrobial peptides and pro-inflammatory cytokines by polynuclear neutrophil cells. IL-17A, IL-17F, IL-22, and IL-21 are the effector cytokines produced by Th17 cells; IL-23 is mainly produced by myeloid cells (macrophages), dendritic cells, and keratinocytes and favor expansion and stabilization of Th17 responses.

Biologically active IL-23 consists of IL-23p19 linked *via* a disulfide bond to IL-12p40 and signals through the IL-23R in complex with IL-12Rβ1 (9, 10). The co-localization of IL-23R and IL-12Rβ1 enables the complex to activate Janus kinase 2 (JAK2) and tyrosine kinase 2 (10), which subsequently phosphorylates signal transducer and activator of transcription 3 (STAT3) (10, 11). The phosphorylation of STAT3 leads to its translocation into the nucleus and further activates the transcription factor retinoic acid-related orphan receptor gamma t (RORγt). RORγt expression induces the transcription of downstream cytokines IL-17A, IL-17F, and IL-22 (12). RORγt also induces the expression of the chemokine receptor CCR6, which allows for the migration of Th17 in inflamed tissues. The binding of CCL20 on CCR6 allows for the chemoattraction of dendritic cells, effector and memory T cells and B cells, especially on the mucosal surface in homeostatic and pathogenic conditions (13). The IL-23 pathway induces a positive feedback loop able to maintain the pathogenic activity of this pathway (14).

IL-17A was cloned in 1993 and was considered the IL-17 family leader, but other proteins structurally related to IL-17A were further identified in the 2000s. Thus, the IL-17 family consists of IL-17A, IL-17B, IL-17C, IL-17D, IL-17E, and IL-17F. IL-17A is mainly produced by Th17 cells. IL-6 and transforming growth factor β (TGFβ) promote the initial differentiation of Th0 to Th17 cells, whereas IL-23 stabilizes and expands Th17 cells in mice (15). The activity of IL-17A is mediated *via* a heterodimeric receptor consisting of IL-17RA and IL-17RC. This complex recruits the nuclear factor κB (NF-κB) activator 1 (ACT1) adaptor protein to activate several pathways such as mitogen-activated protein kinases (MAPKs) including p38 MAK, c-jun N-terminal kinase (JNK), extracellular signal-regulated kinase (ERK), JAK, STAT, and phosphoinositol 3 kinase (PI3K). It also induces several pro-inflammatory cytokines (IL-1β, IL-6, tumor necrosis factor α [TNFα], C-C motif chemokine ligand 2 [CCL2]), antimicrobial peptides (β-defensin), and matrix metalloproteinases [reviewed in (16)].

**Figure 1:**
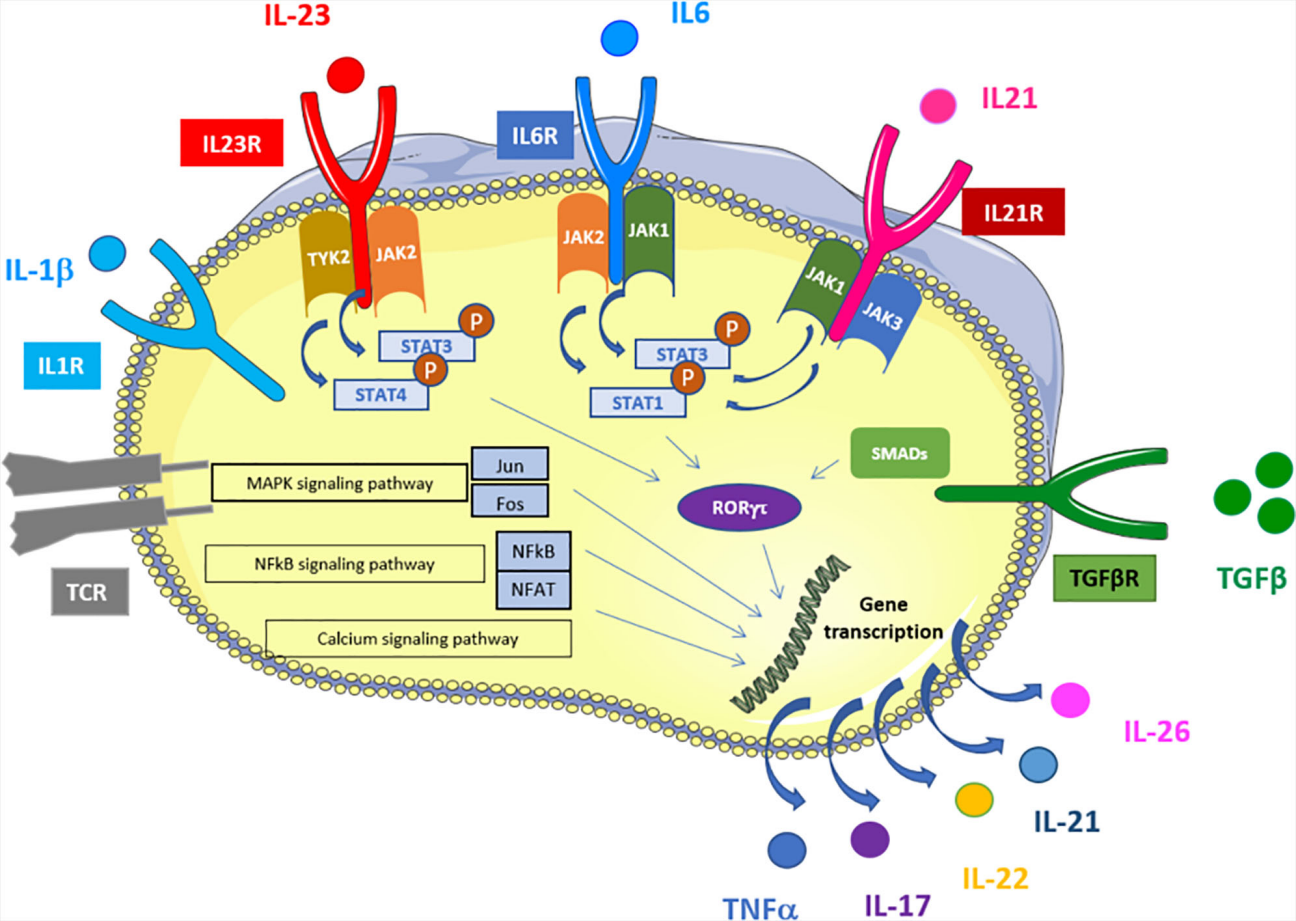
Schematic representation of signaling and transcriptional regulation of Th17 polarization. Th17 cells are induced upon TCR activation in the presence of TGFβ, IL-1β, IL-6, IL-21, and IL-23. IL-6, IL-21 and IL-23 activate several STATs that bind to the promoter regions and activates transcription of RORγt. In addition, IL-1 induces RORgt via P38/mTOR and IRF4. TGFβ stimulation induces RORγt activation via SMADs. RORgt leads to IL-17 and other Th17-related cytokines expression. TCR activation activates MAPK, NFkB and calcium signaling pathways that also induce Th17-related cytokines expression via alternative transcription factors (NFAT, NFkB, Jun and Fos). Adapted from KEGG pathways https://www.genome.jp/kegg-bin/show_pathway?hsa04659.

**Figure 2:**
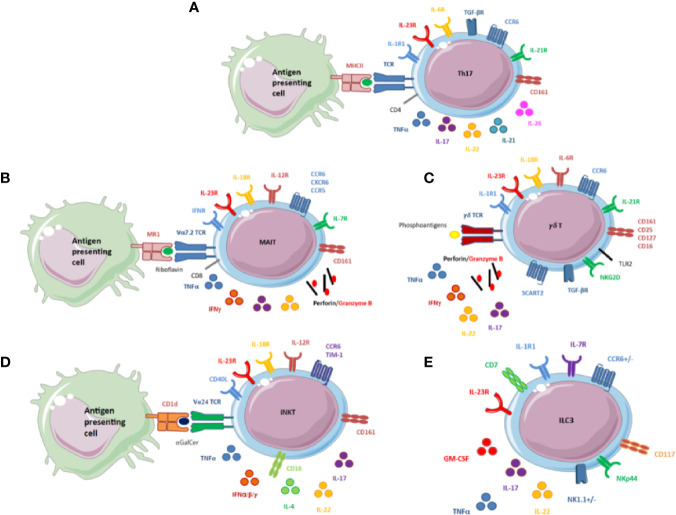
**(A)** Schematic overview of the main receptors and secreted cytokines of Th17 cells. CD4+Th17 cells are induced on T-cell receptor (TCR) activation in the presence of TGFβ, IL-1β, IL-6, IL-21, and IL-23. IL-6, IL-21, and IL-23 activate STAT3, which binds to the promoter regions and activates transcription of RORγt and IL-17. Th17 cells also produce other cytokines, mainly pro-inflammatory cytokines: TNFα, IL-21, IL-22, and IL-26. Th17 cells possess 2 markers specific to IL-17 producing cells: CD161, a marker of activation belonging to the C-lectin family, and CCR6, a chemokine receptor that binds only one chemokine, CCL20. It may regulate the migration and recruitment of dendritic cells and T cells during inflammatory and immunological responses. **(B)** Schematic overview of the main receptors and secreted cytokines of mucosal-associated invariant T (MAIT) cells. MAIT cells express an invariant TCR, Vα7.2. They recognize the conserved major histocompatibility complex-like protein 1 (MR1), which presents a bacterial-derived ligand, riboflavin. MAIT cells express various cytokines and receptors. In the cytokine environment, MAIT cells can release cytotoxicity granules, perforin and granzyme **(B)** They also display specific receptors to induce a type 1 immune response (IFN, IL-12, and IL-18 receptors to produce TNFα and IFNγ) and a type 3 immune response (IL-23R to produce IL-17 and IL-22). IL-7 receptor (IL-7R) is also displayed on the surface of MAIT cells. IL-7R plays a critical role in the development of immune cells and could be of particular interest for these cells. MAIT cells possess the 2 markers specific to IL-17–producing cells, CD161 and CCR6. CXCR6 and CCR5 are also expressed, but their roles are not fully understood. **(C)** Schematic overview of the main receptors and secreted cytokines of γδ T cells. γδ T cells express a TCR that does not engage major histocompatibility complex antigen complexes but rather conserved phosphoantigens of bacterial metabolic pathways. Self-induced proteins overexpressed by infected cells or tumor cells are detected by NKG2D expressed at the surface of γδ T cells. IL-1, IL6, IL-18, IL-23R, and TGFβ induce the production of IL-17. TLR2 is expressed at the surface of these cells and could also be involved in the production of IL-17 by γδ T cells. γδ T cells can also release cytotoxicity granules: perforin and granzyme B and other pro-inflammatory cytokines, IL-22, TNFα, and IFNγ. IL-7 receptor (IL-7R) is on the surface of γδ T cells and participates with CD25 and SCART2 in maintenance of the phenotype. γδ T cells possess the 2 markers specific to IL-17–producing cells, CD161 and CCR6. **(D)** Schematic overview of the main receptors and secreted cytokines of invariant natural killer T (iNKT) cells. iNKT cell activation depends on the interaction between the invariant TCR antigen Vα24 with CD1d loaded with the prototypic antigen glycosphingolipid a-galactosylceramide (αGalcer). iNKT cells can also be activated independently by the cytokine environment. iNKT cells display different receptors: “type 1”: IL-12 and IL-18 receptors”; type 3”: IL-23 receptor; but also CD40L to interact with B cells. This combination allows for releasing various cytokines including TNFα, IFNα, IFNβ, IFNγ, IL-4, IL-17, and IL-22. iNKT cells also possess 2 markers specific to IL-17–producing cells, CD161 and CCR6. **(E)** Schematic overview of the main receptors and secreted cytokines of innate lymphoid cells (ILCs). ILCs are characterized by lack of markers specific for T cells, B cells, and other hematopoietic cells. ILC development depends mainly on IL-7. ILCs are tissue-resident cells and possess chemokine receptors for migration, CCR6 for ILC3. ILC3s are related to RORγT and produce IL-17, IL-22, TNFα, and granulocyte macrophage–colony-stimulating factor in response to IL-1β and IL-23. Human ILC3s are identified by the combination of NKp44 that may normally contribute to the increased efficiency of activated natural killer (NK) cells; CD117, a tyrosine kinase receptor, and CD127 (IL-7Rα).

IL-21 and IL-22 are two other key cytokines secreted by Th17. IL-22 has a protective effect on the cutaneous, digestive, and respiratory-tract barriers *via* the production of anti-bacterial proteins and chemokines, the increase in cellular mobility, and the expression of molecules amplifying its action. IL-22 can act synergistically with TNF and appears to enhance the effect of IL-17A and IL-17F in some *in vitro* models [reviewed in (17)]. The other sources of IL-22 are somewhat like those of IL-17A (type 3 innate lymphoid cells [ILCs] mainly and invariant natural killer T [iNKT] cells) *via* RORγt. However, Th1 lymphocytes produce IL-22, with level correlated with interferon γ (IFNγ) and T-bet levels. Some authors have even described an independent population named Th22. The production of IL-22 goes through the transcription factors aryl hydrocarbon receptor (AhR) and RORγt as for Th17 (but with induced IL-22 mRNA expression less important for the latter). These results suggest that differentiation to either of these two cell types relies on RAR Related Orphan Receptor C (RORC) expression [reviewed in (17) and (18)]. IL-21 is also produced by Th17 and has an autocrine action. Even if not mandatory for Th17 differentiation, IL-21 allows for the stabilization of the Th17 phenotype and proliferation capacities. IL-21 increases the expression of IL-23R and induces the expression of RORγt [reviewed in (19) and (20)] (**Figures 1** and **2A**).

## Alternative Sources of IL-17: Innate Immune Cell Subsets

Several groups are currently investigating whether these innate immune subsets are dysregulated in SpA and to what extent they might contribute to disease pathogeny.

### Mucosal-Associated Invariant T (MAIT) Cells

Various cytokines are expressed by MAIT cells, such as IFNγ, IL-17, or cytotoxicity granules after a large pattern of stimuli (21, 22). The cells display an effector phenotype with chemokine receptor expression suggesting their ability to migrate in tissues. MAIT cells express an invariant T-cell receptor (TCR; Vα7.2) activated by the major histocompatibility complex class I-related protein 1 (MR1) (**Figure 2B**). MR1 is ubiquitously expressed by many cell types, especially hematopoietic and epithelial cells, so they can act as antigen-presenting cells (23). At the exit of the thymus, MAIT cells are still naive cells but very quickly become memory cells after interacting with B cells and the commensal flora (24). Indeed, B cell-deficient patients lack MAIT cells. The mechanism of interaction and the stage of maturation at which this interaction occurs is not known, but the ability of B cells incubated with bacteria to induce MAIT cells suggests an interaction with MR1 (25, 26). The transcription factors promyelocytic leukaemia zinc finger protein, RORcT, and CD161 are also rapidly expressed so that once in the periphery, MAIT cells acquire a memory phenotype (26).

These cells are related to the CD8 cell subset. In fact, they express CD8αα homodimers or intermediate levels of CD8αβ or are double negative for CD8 α and β chains (27). MAIT cells are mainly present in the liver, where they represent up to 20% to 50% of T cells (28, 29), but they are also found in blood, mesenteric lymph nodes, lamina propria, and especially inflammatory tissues during different diseases. Indeed, the presence of chemokine receptors (CCR5, CCR6, CXCR6) suggests a tissue tropism, and the presence of cytokine receptors such as IL-23R and IL-18R is linked to their ability to secrete pro-inflammatory cytokines, mostly TNFα, IFNγ, and IL-17, after strong stimulation (28–30). However, stimulation with CD3/CD28 or antigen-presenting cell infection is sufficient to induce IFNγ, whereas PMA/ionomycin is necessary to induce IL-17A (21, 22, 31–34).

### Invariant Natural Killer T (iNKT) Cells

iNKTs were first described for their regulatory role in oncology and autoimmunity. On the one hand, they promote cell-mediated immunity against tumors and bacteria or viruses, and on the other, they limit cell-mediated immunity and allograft rejection. This tolerogenic role in autoimmunity has been described in different experimental models such as type 1 diabetes, multiple sclerosis, systemic lupus erythematosus, rheumatoid arthritis (35); ozone-induced asthma (36); and collagen-induced arthritis (37), but there are discrepancies with opposite results depending on the treatment protocol or mouse strains.

In humans, these cells represent approximately 0.01% of peripheral blood mononuclear cells, with considerable inter-individual variability (approximately 100-fold). The iNKTs have a Vα24-Jα18-Vβ11 invariant TCR (loading lipids or glycolipids) and are CD1d-restricted (**Figure 2D**). Upon activation, they can release a large panel of pro- and anti-inflammatory cytokines. The combination of the type of molecules loaded on CD1d, the binding kinetics, and the signal strength determine cell polarization and the profile of pro- or anti-inflammatory released cytokines (38).

A small subpopulation of IL-17A–producing iNKTs has been described in mice. These IL-17^+^ iNKT cells express the RORγT transcription factor IL-23R and the chemokine receptor CCR6 (39–42). iNKT cells are found in the thymus, spleen, liver, and lungs and are highly enriched in peripheral lymph nodes. In vivo, they can produce high concentrations of IL-17, within 2 to 3 hr after stimulation with lipopolysaccharide or lipopolysaccharide-activated dendritic cells [reviewed in (43)]. Recently, this specific population has been described in humans. The RORγT^+^ iNKT subset of cells accounted for 2.1% of the parent population and produced IL-17A and IL-22. The IL-23R was not expressed on the surface of these cells, but the authors found IL-23R mRNA expression, which suggested that the expression of the receptor on the cell surface depended on the inflammatory context. Secretion of IL-17A needed the combined presence of IL-23 with invariant TCR Vα24 stimulation (44).

### Gamma delta T Cells (γδ T Cells)

γδ T cells represent approximately 3% to 5% of all lymphoid cells found in blood (45) and 50% of the total intraepithelial lymphocyte population at mucosal and epithelial sites, especially in the gut. In contrast to αβ T cells, whose mode of action is based on the TCR (46), γδ T cells are sensitive to a variety of antigens (47), mainly phosphorylated metabolites, also called phosphorantigens, issuing from bacterial metabolic pathways (48). γδ T-cell differentiation is already advanced at their exit from the thymus, which limits their plasticity in the periphery. Thus γδ T cells are activated by the cytokine environment rather than their TCR. Differentiation and expansion of IL-17^+^ γδ T cells would depend mainly on IL-7 and TGFβ. We can now distinguish at least 4 subpopulations of Tγδ lymphocytes based on their effector function: IL-17 producers, IFNγ producers, innate-like Tγδ (49), and γδ T regulatory cells described in 2009 by Otsuka et al., only in mouse (50). γδ T cells express chemokine receptors, cytokine receptors, and pattern recognition receptors, and the receptors have been found involved in activating γδ T cells, especially IL-17 (**Figure 2C**). γδ T cells possess receptors for IL-1, IL-6, IL-18, IL-23, and TGFβ1 promoting IL-17 production. Signaling pathways traditionally involved in IL-17 production are also described for γδ T cells: Toll-like receptor 2 and Dendritic Cell-associated C-type lectin 1 (dectin 1), as well as the internal AhR. Recently, other types of interaction have been found involved in the production of IL-17A: CD30/CD30L and CD27. B- and T-lymphocyte attenuator and Notch could regulate this production by inhibiting RORγT (51). In addition, models of IL-2– and IL-25–deficient mice have shown that IL-2 plays a key role in maintenance of IL-17A–producing cells. The IL-2 receptor α chain (CD25) but not β chain (CD122) is expressed on the surface of IL-17–producing γδ T cells (52). IL-17^+^ γδ T cells share many phenotypic characteristics with Th17, particularly STAT3 and RORγT transcription factors as well as surface markers CCR6 and IL-23R. The cytokine environment mainly determines the differentiation and expansion of this IL-17–producing subpopulation of γδ T cells: IL-7, whose receptor IL-7Rα is more expressed in the CD27^-^ cell fraction, and TGFβ, whose role seems to amplify the production of IL-17 (53). The role of the TCR on γδ T-cell effector functions is still debated.

### Innate Lymphoid Cells (ILCs)

ILCs are divided into 4 groups according to the specific cytokines they produce and the specific transcription factors they express according to their profile of differentiation and function. ILCs are tissue-resident innate immune cells involved in the host defense against pathogens and in tissue remodeling. Group 1 ILCs (ILC1s) are defined by their expression of T-bet and the production of IFNγ and TNFα. ILC1s have cytotoxic properties and are mainly found in the intestine, lung, and skin. Group 2 ILCs (ILC2s) express GATA3 and type 2 cytokines (IL-5, IL-9, and IL-13) upon IL-33 and IL-25 stimulation. ILC2-secreted cytokines can promote M2 macrophage polarization. ILC2s reside in lung, intestine, gut, and skin. Group 3 ILCs (ILC3s) and lymphoid tissue inducer (LTi) cells both express RORγt and produce IL-17 and/or IL-22 (**Figure 2E**). LTi cells drive the formation of secondary lymphoid structure such as lymph nodes and Peyer’s patches during fetal development. ILC3s are found in skin and are particularly in psoriasis lesions (54). ILC3s seem to be critical for gut homeostasis by modulating cell proliferation, cytokines and antimicrobial peptide production, permeability of the intestinal barrier, and interactions between microbiota and CD4^+^ T cells. NKp44^+^ ILC3s are found in the mouse intestine and have a protective effect against induced colitis with a “homeostasis keeper” function *via* IL-17 secretion (55, 56). In healthy individuals, 0.01% to 0.1% of circulating lymphocytes express a CD127^+^ ILC phenotype. Recently, Lim et al. showed that most CD127^+^ ILCs found in peripheral blood are ILC2s, with near absence of NKp44^+^ ILC3s (57) and CD127^-^ ILC1s (58). The authors also showed that ILC subpopulations differentiated in tissues and persisted in blood as a precursor. In the human gut, ILCs are mainly represented by CD127^+^ ILC1s that are involved in the defense against pathogens in response to danger signals (58).

In humans, different subpopulations with similar phenotypic characteristics can be found on the same site, which suggests a plasticity of these cells. The cytokine environment is at the origin of the trans-differentiation of ILC3s to ILC1s: the pro-inflammatory cytokines IL-12 and IL-18 induce a downregulation of RORγT associated with an upregulation of T-bet (59, 60). This type of trans-differentiation has been observed in Crohn’s disease (61).

### Neutrophils

Neutrophils are the first line of defense of the immune system, constituting a cellular barrier against fungi and bacteria but also against altered endogenous cells or molecules. Neutrophils are mature cells that are rapidly activated and functional but with a short lifespan (a few hours) (62). The essential immune functions and short lifespan of neutrophils demand their constant production in bone marrow, called granulopoiesis. This highly regulated mechanism produces 10^11^ neutrophils each day (63). As a first-line defense, neutrophils have various functional capacities that occur alone or in combination (64, 65) and allow for destruction of the pathogen: phagocytosis, oxidative stress, release of cytokines, and NETosis. Furthermore, neutrophils are able to interact with immune cells, favoring the maturation of dendritic cells and natural killer cells and Th1 and Th17 differentiation (66). Conversely, Th17 cell-derived cytokines (e.g., IL-17, CXC-chemokine ligand 8 [CXCL8; also known as IL-8], IFNγ, TNF, and granulocyte macrophage–colony-stimulating factor [GM-CSF]) favor recruitment, activation, and prolonged survival of neutrophils at inflammatory sites. Cellular interactions between lymphocytes and neutrophils are crucial. These exchanges participate in regulating the adaptive immune system as suggested by the migration of neutrophils to lymph nodes. Nevertheless, neutrophils have limited transcriptional capacity. The quantity of mRNA produced corresponds to only 5% of that of the other leucocytes (67). However, considering that neutrophils recruited to inflamed tissues greatly outnumber other leukocytes, the overall impact of neutrophil-derived cytokines in the inflammatory response could counterbalance this transcriptional limitation. Like other cell populations, sub-populations of neutrophils with very specific abilities seem to exist both under homeostatic and pathological conditions.

Granulopoiesis is finely regulated by GM-CSF. This phenomenon induces a release of other pro-inflammatory factors (such as cytokines, chemokines, and matrix metalloproteinases) by mesenchymal and myeloid cells, thus allowing neutrophil recruitment and activation: the “neutrostat” system (68). The direct production of IL-17A by neutrophils is a highly controversial subject. Taylor et al. provided evidence that neutrophils could produce IL-17A not only for auto-loop activation but also to amplify the phenomenon of oxidative burst and defense against fungi. After preincubation with a fungal stimulus and incubation in the presence of IL-6 and IL-23, IL-17A mRNA and protein expression by neutrophils was detected. The neutrophils expressed IL-23 and IL-6 receptors on their cell membrane. The authors then showed that IL-17A production depended on RORγt and Dectin2. However, the cytokine concentrations used to stimulate the neutrophils were very high, far from “physiologic” conditions (69). More recently, a work from Tamassia et al. failed to reproduce these results. The authors showed with multiple methodological approaches that even with the same stimulations, neutrophils could not express or induce secretion of IL-17A at any stages of maturation. They also demonstrated that the antibodies used for immunohistochemistry/immunofluorescence were not specific to IL-17A and could induce false positive results. Finally, they showed the lack of histone marks associated with active and poised regulatory elements at the IL-17A locus of neutrophils as compared with Th17 cells, which suggested the inability of neutrophils to express IL-17A mRNA (70). A recent work from our group also confirmed that stimulated neutrophils from SpA patients were unable to express IL-17 A or IL-17F, both at the mRNA and protein levels (personal data).

### Mast Cells

Mast cells are innate tissue resident cells of the immune system. They are not fully differentiated and are able to survive months or years despite not circulating in their mature form. Mast cells possess lysosome-like secreting dense granules in their cytoplasm that are released upon activation (71, 72) and represent a unique array of immune-modulating molecules. The activation signals rely on various stimulation processes including IgE receptor crosslinking, complement activation, neuropeptides, and toxin stimuli. Activation of mast cells induces the exocytosis of pre-formed molecules stored in granules. Mast cells “communicate” with various cell types, including cells belonging to the innate and adaptive immune system such as lymphocytes, macrophages, dendritic cells, and neutrophils (73). In several mouse models, mast cells participate in neutrophil recruitment *via* IL-8 (74): synovium in collagen-induced arthritis (75), skin in bullous pemphigoid (76), and meninges in experimental autoimmune encephalomyelitis (77). The mast cell ability to produce IL-17 was first suggested in atherosclerosis. Indeed, carotid endarterectomy immunohistochemical analysis revealed IL-17A/F^+^ mast cells in complicated plaques, with no observation of IL-17A/F^+^ T cells (Th17 cells). The ability of mast cells to produce IL-17A has also never been confirmed in mice models. A recent work involving tonsil biopsies and synovial tissue suggested that mast cells were able to capture circulating IL-17A and release it *via* a dynamic mechanism of endo- and exocytosis (78). The authors demonstrated that mast cells did not possess the necessary transcriptional machinery allowing for IL-17A synthesis. Even after stimulation, they did not find mRNA for IL-17A, whereas in immunofluorescence assessment of tonsillar sections, IL-17A was present in the cytoplasm of mast cells within secretory vesicles. Dynamin 2 GTPase appeared to be the process *via* which IL-17A internalization occurred, a mechanism independent of IL-17A receptors. The externalization process was found only indirectly, and unlike cytokines classically released by mast cells, only the release of IL-17A used this process (78).

### Eosinophils

Eosinophils display bi-lobed nuclei and specific granules characteristic of this cell population compared to the other granulocytes (neutrophils and basophils). Eosinophils are specifically involved in type 2 immunity. They are mainly increased in response to helminth infection and in the context of allergic disease. Eosinophils are able to enhance the immune responses mediated by T helper cell type 2 (Th2) through the production of IL-4 and by acting as antigen-presenting cells. Eosinophils produce chemoattractants for DC and effector Th cells such as CCL17, CCL22, CXCL9, CXCL10, and Eosinophil-derived-neurotoxin (EDN) [reviewed in (79)]. Several teams reported a production of IL-17 by eosinophils in specific inflammatory conditions. Shimura et al. showed that IL-17A—but not IL-17F—was crucial in LPS-induced sepsis in mice (80). Eosinophils were also able to produce IL17A after monosodium urate crystals stimulation (81). Other groups showed that eosinophils were able to produce IL-17A in response to Aspergillus fugimatus together with IL-23, thus contributing directly to the modulation of the IL23/IL17 axis (82, 83). To date, the capacity of IL17 production by eosinophils has not been demonstrated in immune mediated inflammatory diseases.

## The Role of IL-17 in SpA: From Animal Models to Human Disease

Sherlock et al. used the mini circle DNA technology with IL-23 overexpression to induce an “SpA-like” phenotype with enthesitis in B10 RIII mice. Bone remodeling was associated with increased expression of IL-17A and IL-22, the latter even more important for bone formation than IL-17A.

Another group used the SKG mouse model (housed under specific pathogen conditions) injected with β1,3-glucan to obtain a phenotype closer to axSpA. The SKG strain develops spontaneous IL-17-dependent autoimmune inflammatory arthritis under microbial conditions induced by pulmonary fungal infection. β-glucan is a component of fungal cell walls including Candida and Aspergillus. This combination leads to axial and peripheral arthritis in mice. From these results, spondylitis was IL-23–dependent, as was arthritis and ileitis (84, 85).

The HLA-B27 transgenic rat is a classical model of SpA with axial and peripheral arthritis, nail dystrophy, gut inflammation, and orchitis/epididymitis (86). Th17 cells are involved in this rat model of SpA (87). HLA-B27 transgenic rats immunized for *Mycobacterium tuberculosis* is another model suggesting that the onset of the SpA phenotype could depend on IL-23. Indeed, anti-IL-23R treatment used before the appearance of the symptoms prevented the development of the axial disease in these rats but not the same treatment injected after the appearance of the symptoms (88). These data, if translatable to human disease, would suggest that axSpA should be IL-23–dependent in the preclinical disease phase but IL-23–independent once the disease is established. The same group showed that fibroblast-like synoviocytes exposed to IL-17A differentiated into osteoblasts. Using the same rat model with anti-IL17A treatment, axial inflammation (spondylitis) decreased (albeit not significantly). Blocking IL-17A appeared to limit bone remodeling and especially the periosteal new bone formation and to reduce peripheral and axial inflammation (89).

In humans, genome-wide association studies have shed light on the IL-23/IL-17 axis: 6 of the 48 non-MHC loci are genetically associated with SpA-involved genes in this pathway (RUNX3, IL-23R, IL-6R, IL-1R2, IL-12B, tyrosine kinase 2) (90). These data suggest that the inflammatory response in SpA may result from a complex interaction between different immune cell types and the key role of the IL-23/IL-17 axis in chronic inflammation.

Appel et al (7). assessed the facet joints of axSpA patients undergoing a surgical procedure. Immunostaining of histological sections revealed that IL-17–producing cells were mainly neutrophils and, in smaller proportions, T lymphocytes. Of note, the study population included patients with advanced disease, requiring surgery.

Several groups have reported an increased proportion of Th17 in the peripheral blood of AS patients as compared with controls or patients with other inflammatory conditions. Other IL-17–secreting cells are increased in number in AS patients. In peripheral blood, a study reported a three-fold higher frequency of circulating γδ T cells and five-fold higher frequency of IL-23R–expressing γδ T cells in AS patients versus healthy controls and versus rheumatoid arthritis patients, respectively (8). In this study, γδ T cells were suggested to be the dominant IL-17 producers in AS.

Another study involving AS patients reported decreased number of MAIT cells in peripheral blood but increased subpopulation of IL-17^+^ MAIT cells as compared with controls. In this study, MAIT cells appeared to concentrate in the synovial fluid, thus suggesting a migration of these cells to the inflammatory sites. MAIT cells produced large amounts of IL-17A under IL-7 stimulation but surprisingly not IL-23 stimulation (91). This increase in IL-17A^+^ MAIT cells was recently confirmed in a cohort with exclusively axSpA (92).

## The Role of IL-17 in Bone Formation

Inflammatory and painful entheses (the sites of attachment of tendons, ligaments, fascia, or joint capsules to bone) are the distinctive pathological features of SpA. Bone formation in SpA is closely linked to the inflammatory processes at the spinal entheses. Periosteal appositions at the sites of past inflammatory entheses are observed, constituting the basis for bone formation. This ossification goes through several stages: apoptosis of chondrocytes, which are further replaced by osteoblasts into osteocytes partitioned in the matrix (93, 94). Nevertheless, the mechanisms leading to bone formation at the inflamed entheseal sites in SpA are not fully understood.

Among other hypotheses, mechanical stress could be a key trigger of entheseal inflammation and further new bone formation. This hypothesis was assessed in the TNF^ΔARE^ mouse model in which chronic and deregulated TNF production leads to axial and peripheral arthritis associated with a Crohn’s-like ileitis. In this TNF-driven mouse model of SpA, Erk1/2 signaling plays a crucial role in the mechanical stress-induced inflammation. New bone formation was strongly promoted at entheseal sites by biomechanical stress and was correlated with the degree of inflammation.

At the entheseal level, IL-17 amplifies the inflammation by promoting the secretion of pro-inflammatory cytokines by the resident mesenchymal cells. GM-CSF, IL-6, IL-8, and IL-17 are chemo-attractants for neutrophils contributing to activate the inflammatory loop (95–98). The cytokine micro-environment seems to be determinant in bone remodeling phenomenon. IL-17 combined with TNF increases calcified matrix formation from mesenchymal cells when they are exposed to conditions leading to bone formation (99). Ono et al. showed a direct role of IL-17A on bone healing after a fracture: there was an increase in IL-17A at the fracture site enhancing bone regeneration. IL-17A activated osteogenesis by differentiating the mesenchymal cells present at the fracture site. Osteoclastogenesis was not affected. The major source of IL-17A was γδ T cells and in particular Vγ6 (100). These data were confirmed *in vitro* by Osta et al (99). However, the authors pointed out that this osteogenic differentiation of mesenchymal cells was only possible by combining IL-17A and TNF, IL-17A alone having no effect (99). Moreover, the combination of these two cytokines induced a decreased expression of DKK1 and RANKL in mesenchymal cells, thus contributing to an increased osteogenesis. The authors suggested that the cellular environment (i.e., the presence or absence of osteoclasts) crucially determined the effect of these two cytokines: the presence of mesenchymal cells combined with the absence of osteoclasts at the entheseal level could explain why IL-17A and TNF could both contribute to bone formation in SpA (99).

## The Role of Innate Immune Cells Secreting IL-17: A Path to Understanding the Failure of IL-23 Blocking Agents in axSpA?

Sherlock et al. demonstrated that the overexpression of IL-23 in mice induced a SpA-like phenotype with enthesitis but not requiring a mechanical overload. They identified a specific subset of enthesis-resident T cells: CD3+, CD4, and CD8 double-negative and expressing IL-23R. These cells, upon IL-23 stimulation, secreted high amounts of IL-17 and IL-22, inducing typical features of SpA, such as enthesitis and new bone formation (95). Another study using the same mouse model identified these cells as γδ T cells (101). More recently, Cuthbert et al. investigated the presence of γδ T cells in normal axial entheses harvested during orthopedic procedures from patients with mechanical back pain (e.g., osteoarthritis or scoliosis). Two γδ T-cell subsets were identified (γδ1 and γδ2). The γδ1 subset lacked IL-23R transcripts but was able to express IL-17A upon PMA/ionomycin or CD3/CD28 stimulation. Neither Il-17A nor IL-17F transcripts were expressed upon IL-23 stimulation. These findings might suggest that γδ1 T cells could be involved in IL-17 production in spinal enthesis from SpA patients, independent of IL-23. This could be an attractive explanation for the failure of IL-23 blocking treatments in axial SpA (102). Using a similar approach on patients undergoing a surgical procedure, Cuthbert et al. reported the presence of ILC3s in spinal entheses, with the same characteristics as ILC3s collected from synovial fluid from SpA patients [i.e., IL-23R, STAT3, and RORγt transcript expression (103)]. IL-17A transcript expression by these cells was obtained on IL-23/IL-1β stimulation.

Gracey et al. showed an increased number of IL-17^+^ MAIT cells in peripheral blood from AS patients as compared with controls (91). These cells were able to express IL-17A upon priming with IL-7 but not IL-23 stimulation. IL-17A expression was assessed by flow cytometry.

How do these results fit into the current context of clinical trials showing failure of drugs targeting IL-23 in axSpA and the successful approaches of IL-17A targeting drugs? Obviously, blocking the terminal cytokine IL-17A is a valuable approach in axSpA, whatever the source of production: Th17 cells, MAIT cells, γδ T cells, or ILC3s. In contrast, IL-23 blockade is not effective for axial disease, which suggests that cells involved in the axSpA pathogenesis might be able to express IL-17A independent of IL-23. Preliminary results from several groups suggest that MAIT cells and γδ1 T cells could be the culprits, with the proviso that the said studies were conducted in healthy individuals.

## Conclusion

**Figure 3:**
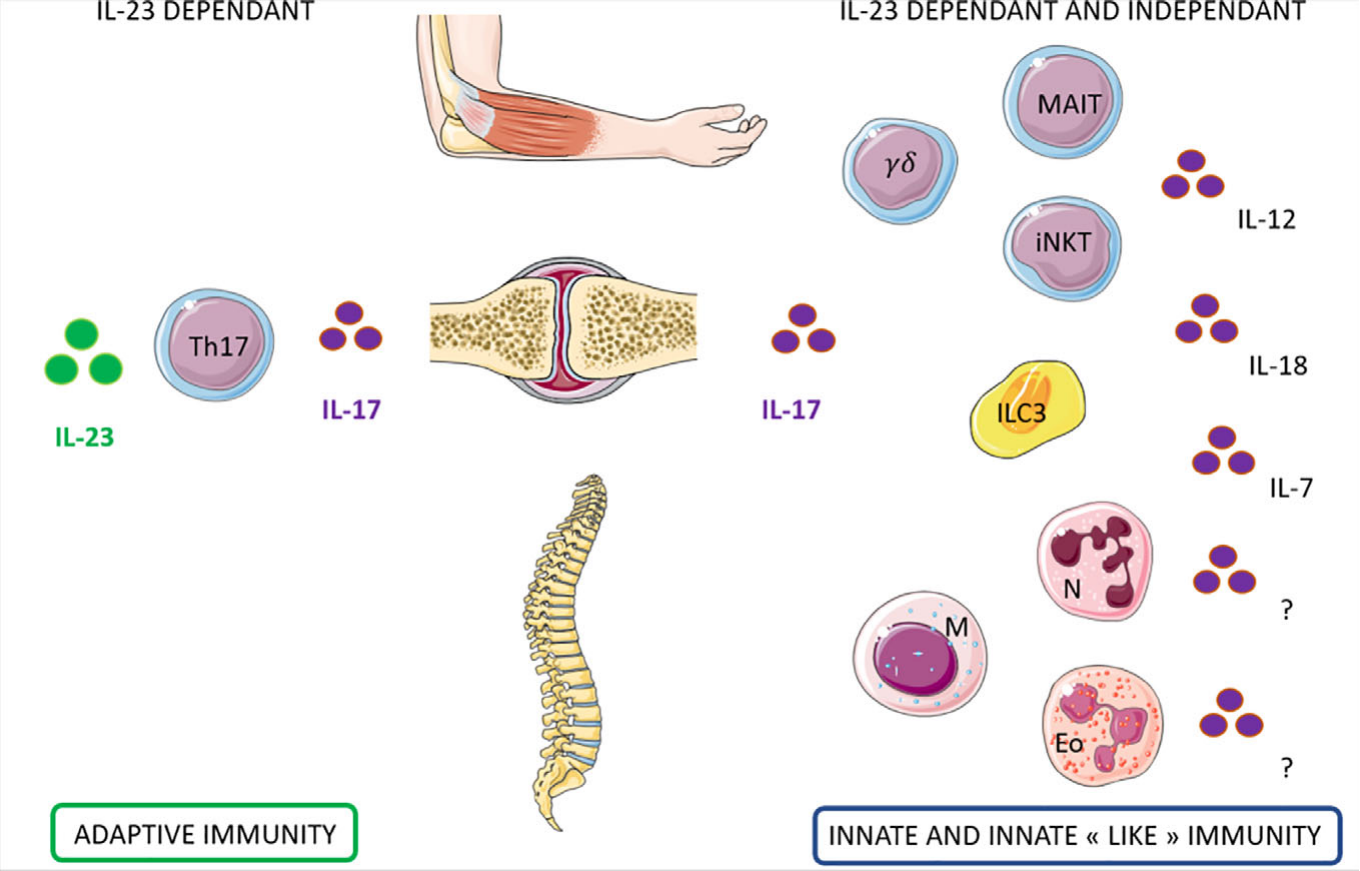
Candidate cells that may contribute to IL-17 production dependent and independent of IL-23 in Spondyloarthritis. **Left:** Th17 cells contribute to the production of IL-17 through IL-23 dependant pathway for the entheseal and the peripheral involvement of Spondyloarthritis. **Right:** Innate immune cells may contribute to the production of IL17 through IL-23 but also independently. Several lymphoid cells (MAIT, γδT cells, iNKT, and ILC3) and myeloid cells (neutrophils, mast cells, eosinophils) has been identified as potential candidates. The cytokines that could induce this production independently from IL23 are still under investigation.

In this review, we have described the different IL-17–producing cells belonging to the innate immunity compartment. Lymphoid cells (iNKT cells, MAIT cells, γδ T cells, ILC3s) can produce IL-17 *via* engagement of their TCR. Nevertheless, these cells also possess a unique ability to produce IL-17 independent of the conventional TCR–antigen interaction, in response to their cellular and cytokine/chemokine environment. Thus, they have dual IL-17 production capacity that could be of prime importance in SpA pathogenesis. In addition, their ability to secrete IL-17 is immediate, not requiring differentiation or proliferation steps. They are at the ultimate stage of maturation and thus ready to “draw.” Finally, as for other cells belonging to the innate immunity, their migratory capacity is also pronounced. These cells are found in greater quantity on all inflammatory sites, which suggests that they may be responsible for IL-17–driven inflammation in target tissues of SpA (i.e., spine, skin, gut, or joints). Their precise role in the pathophysiology of SpA remains to be better defined, but preliminary results from several teams converge to suggest their major importance in the pathophysiogenesis of SpA, especially MAIT cells and γδ1 T cells that are able to express IL-17A upon priming with various cytokines but independent of IL-23 (**Figure 3**). These results might be a path to understand why IL-17A blocking agents are effective in axSpA in contrast to IL-23–blocking drugs. These clinical results are a nice example of a reverse path from bedside to bench, where the results of therapeutic trials make us reflect more in depth on the pathophysiology of the disease.

## Author’s Note

Servier medical art was used for the realization of the figures.

## Author Contributions

NR and CM-R contributed equally to this work and approved it for publication. All authors contributed to the article and approved the submitted version.

## Funding

NR was funded by a Poste d’accueil APHP and a grant from Société Française de Rhumatologie.

## Conflict of Interest

The authors declare that the research was conducted in the absence of any commercial or financial relationships that could be construed as a potential conflict of interest.
